# Sensitivity of the Quantiferon-Gold In-Tube Assay in Sputum Smear Positive TB Cases in Indonesia

**DOI:** 10.1371/journal.pone.0012020

**Published:** 2010-08-09

**Authors:** Merrin Rutherford, Bachti Alisjahbana, Winni Maharani, Hedy Sampurno, Reinout van Crevel, Philip C. Hill

**Affiliations:** 1 Department of Preventive and Social Medicine, Centre for International Health, University of Otago, Dunedin, New Zealand; 2 Health Research Unit, University of Padjadjaran, Bandung, Indonesia; 3 Bandung Community Lung Clinic, Bandung, Indonesia; 4 Department of Internal Medicine, Radboud University Nijmegen, Nijmegen, The Netherlands; McGill University, Canada

## Abstract

**Background:**

As part of a formal evaluation of the Quantiferon-Gold in-tube assay (QFT-IT) for latent TB infection we compared its sensitivity to the tuberculin skin test (TST) in confirmed adult TB cases in Indonesia. Smear-positive TB disease was used as a proxy gold standard for latent TB infection.

**Methods and Findings:**

We compared the sensitivity of QFT-IT and TST in 98 sputum smear and chest x-ray positive TB cases and investigated risk factors for negative and discordant results in both tests. Both tests showed high sensitivity; (QFT-IT; 88.7%: TST; 94.9%), not significantly different from each other (p value 0.11). Very high sensitivity was seen when tests were combined (98.9%). There were no variables significantly associated with discordant results or with a negative TST. For QFT-IT which particular staff member collected blood was significantly associated with test positivity (p value 0.01). Study limitations include small sample size and lack of culture confirmation or HIV test results.

**Conclusions:**

The QFT-IT has similar sensitivity in Indonesian TB cases as in other locations. However, QFT-IT, like the TST cannot distinguish active TB disease from LTBI. In countries such as Indonesia, with high background rates of LTBI, test specificity for TB disease will likely be low. While our study was not designed to evaluate the QFT-IT in the diagnosis of active TB disease in TB suspects, the data suggest that a combination of TST and QFT-IT may prove useful for ruling out TB disease. Further research is required to explore the clinical role of QFT-IT in combination with other TB diagnostic tests.

## Introduction

Tuberculosis (TB) remains a problem worldwide and, despite significant efforts, mortality and incidence rates remain alarmingly high [1]. Treatment of latent TB Infection (LTBI) has been shown to be an effective and safe method of preventing progression to disease in infected individuals following screening and will need to be part of a TB elimination strategy [2], [3]. Diagnosis of LTBI has historically been by the tuberculin skin test (TST), a test with well-known limitations [4]. Subsequently many developed countries now employ the newly developed Interferon Gamma Release Assays (IGRAs) that show superior sensitivity and specificity for LTBI when compared to the TST [5]. IGRAs evaluate cell-mediated immunity in vitro through the detection of interferon-γ released by TB antigen stimulated T cells [5], [6]. Two such assays are commercially available, QuantiFERON-TB Gold in-tube test (QFT-IT) (QFT-IT test; Cellestis Ltd, Carnegie, Australia) and T-SPOT.TB test (Oxford Immunotec, Abingdon, UK). The QTF-IT includes three antigens, early-secreted antigen target 6 (ESAT-6), culture filtrate protein 10 (CFP-10) and TB7.7, which are absent from all BCG strains and most non-pathogenic mycobacteria [7]. Comparing these tests remains a challenge in the absence of a gold standard. However test performance can be assessed indirectly by employing a gradient of exposure or by using active TB as a proxy for measuring sensitivity to LTBI [8], [9]. The utility of these assays for LTBI diagnosis remain unknown in Indonesia, a country that has the third highest caseload of TB disease in the world [10]. As part of a wider series of studies to indicate performance in the diagnosis of LTBI, we evaluated the sensitivity of a new generation IGRA; QFT-IT in comparison with the TST in newly diagnosed sputum smear positive adult TB cases in Indonesia.

## Methods

### Ethics statement

This study was approved by the Lower South Island ethical committee, New Zealand and the Health Research Unit Ethical Committee University of Padjadjaran, Bandung, Indonesia. QFT-IT kits were donated by Cellestis Australia.

### Study setting and recruitment

Between April 2009 and March 2010 consecutive new TB cases presenting at a community lung clinic in Bandung, Indonesia were screened for inclusion. Eligible patients were: over 15 years of age; sputum smear positive; had radiographic evidence of TB[11] and had taken medication for ≤ one week. Following written informed consent from all participants, demographic and clinical data were collected.

### Procedures

Venous blood was obtained and immediately transferred into the QFT-IT assay kit tubes (nil, mitogen and antigen), vigorously hand shaken then transferred within two hours to a 37°C incubator for 16–24 hours. Incubated samples were centrifuged and stored at 4°C until 28 samples had been collected to allow for batch processing or up until one month after blood collection. The QFT-IT assay was conducted according to the manufacturer's instructions (QFT; Cellestis, Carnegie, Australia). Optical densities were measured using a Biotech® microplate reader and Gentec® software, version 1.08 then imported into QFT-IT specific software (QuantiFERON®-TB Gold analysis software version 2.50.4). Results were interpreted as specified by the manufacturer (QFT; Cellestis, Carnegie, Australia). Immediately after the sample for QFT-IT was drawn a TST was performed by an experienced study nurse on the volar side of the forearm using 2 tuberculin units of purified protein derivative (PPD RT23 Biofarma® Bandung). Induration was measured after 48–72 hrs. A positive result was regarded as ≥10 mm of induration. HIV status was unable to be obtained as testing is not routinely offered to the study population.

### Statistical analysis

Sensitivity with 95% confidence intervals was estimated for each diagnostic test using STATA version 11.0. Factors associated with discordant and false negative results for both tests were identified using bi-variate regression. Test concordance and discordance were estimated using the kappa statistic and McNemar's test respectively.

## Results

Of 152 eligible TB cases 99 (65%) were recruited. The main reason for non-participation was failure to return to the clinic for the study, n = 30 (60%); the next most common reason was that the clinic was considered by the patient too far to return to, n = 4 (8%). Data from one participant were excluded, as he did not receive a TST. Nearly half the patients had 3+ sputum smear status (Table 1). For the QFT-IT 86 patients were positive, 11 (11.2%) were negative and 1 (1%) was indeterminate (Table 2). Excluding indeterminate results the QFT-IT had a sensitivity of 88.7% (95% CI 80.6% –94.2%). If indeterminate results were included the sensitivity was 87.8% (95% CI 79.6% –93.5%). There were 93 TST positive patients (sensitivity 94.9%; 95% CI 88.5% –98.3%). The difference between the sensitivity of each test was not significant (p value 0.11). Excluding indeterminate QFT-IT results, both tests were positive in 84.5%, both negative in 1.0%, QFT-IT positive TST negative in 4.1% and QFT-IT negative TST positive in 10.3% (Table 2). Test agreement was 85.6% (Kappa = 0.06; p value 0.27). Discordance was not significant (p value 0.18). A combination of tests gave a sensitivity of 98.9% (97 of 98).

**Table 1:** Patient characteristics.

Characteristic	Number (%)
**Age (years):**	
Median (Range)	31.0 (15–62)
**Sex:**	
Female	53 (54.1)
**Ethnicity:**	
Sundanese	87 (88.8)
**Time on anti-tuberculosis medication (days)**	
Median (Range)	5.0 (1–7)
**BCG Scar present:**	
No	38 (38.8)
Yes	52 (53.1)
Unknown	8 (8.2)
**Sputum smear status:**	
Three plus	43 (43.9)
Two Plus	21 (21.4)
One Plus	32 (33.3)

**Table 2:** Comparison of results for QFT-IT and TST for sputum smear positive pulmonary TB cases.

	TST result		
Quantiferon Result	Positive	Negative	Total
Positive	82	4	86
Negative	10	1	11
Indeterminate	1	0	1
Total	93	5	98

There were no differences between patients with TST +/QFT-IT - or TST -/QFT-IT + and those with concordant results according to age, sex, ethnicity, presence of a BCG scar, duration of productive cough, sputum smear status and days on treatment with anti-tuberculosis medication (data not shown). For QFT-IT there were no significant differences between groups (true positive vs. false positive results) regarding age, sex, ethnicity, presence of a BCG scar, sputum smear status, duration of symptoms or duration of treatment with anti tuberculosis medication (data not shown). However, test results varied by study nurse; positive test results for each of the three nurses were 16/22 (72.7%), 4/4(100%) and 66/72 (91.6%) respectively; the difference between nurse 1 and 3 of almost 20% was statistically significant (p value 0.01). For the TST no significant differences between those patients who were true positives and those who were false negatives were seen.

## Discussion

In 98 patients with sputum smear confirmed TB, employed as a proxy for LTBI, both the TST and QFT-IT tests displayed high sensitivity (94.9% and 88.7% respectively). A combination of both tests yielded a sensitivity of 98.9%. Our findings show slightly higher sensitivity for both tests than most reports from other developing, high TB burden countries. Among 60 microbiologically confirmed TB patients in India 44 (73%) were positive by QFT-IT [12], in The Gambia of 80 TB culture positive patients 64% were positive by QFT-IT [13] and in South Africa among 131 culture positive TB patients 76% were positive by QFT-IT [8]. It is unlikely that differences are due to varying HIV prevalence among study populations. Variations may be related to differences in health seeking behaviour patterns and study populations[5]: subjects in this study were identified at a community-based clinic and we did not include retreatment or treatment failure patients. TB patients who present relatively early, and with an uncomplicated history, may have less severe disease and less anergy [5].

Interestingly, our data suggest that false negative results from the QFT-IT may be due to procedural issues, as we found negative results to be associated with sample collection personnel. Upon further investigation with personnel we believe that the most likely reason for this difference is how vigorously tubes were shaken. It has previously been shown that levels of shaking vigour affect results [14]. It appeared from observation that nurse 3 tended to shake the tubes more vigorously. A further possibility is differing times to incubation. Recently Herrera et al reported that time to incubation may be a determinant of indeterminate QFT-IT results [15]. However, in this study, all samples were incubated within two hours of collection. The impact of sample collection procedure on QFT-IT results requires further, focused investigation. This study had a much lower rate of indeterminate results for QFT-IT (n = 1, 1%) than other studies [7], [16], [17], [18], which may also be related to processing issues [15]. An emphasis on proper staff training when using this test and prompt incubation is advisable.

This study employed sputum smear positive patients as a proxy for LTBI in lieu of a gold standard, QFT-IT, like the TST cannot distinguish active TB disease from LTBI. In countries such as Indonesia, with high background rates of LTBI, test specificity for TB disease is low [19].Our study was not designed to evaluate the QFT-IT in the diagnosis of active TB disease in TB suspects [17]. However, a combination of TST and QFT-IT may prove useful for ruling out TB disease in some individuals. Further research is required to explore the potential of QFT-IT in combination with other TB diagnostic tests.

With respect to study limitations, we were not able to measure the effect of HIV on test sensitivity, although HIV prevalence within the clinic population is below 2% [20]. Also, despite a relatively large sample size in relation to sensitivity evaluation, our study was probably too small to identify factors important for predicting false negative and discordant test results.

### Conclusion

The QFT-IT is not more sensitive than the TST for detecting active TB in this setting. A combination of QFT-IT and TST offers a sensitivity gain, with further studies required to show whether there is an associated significant specificity loss. Further evaluation of the QFT-IT in Indonesia will assess its performance in child TB case contacts against a gradient of exposure and longitudinal assessment of conversion and reversion rates.
